# Pneumococcal Surface Proteins as Virulence Factors, Immunogens, and Conserved Vaccine Targets

**DOI:** 10.3389/fcimb.2022.832254

**Published:** 2022-05-12

**Authors:** Javid Aceil, Fikri Y. Avci

**Affiliations:** Department of Biochemistry and Molecular Biology, Center for Molecular Medicine, The University of Georgia, Athens, GA, United States

**Keywords:** *Streptoccoccus pneumoniae*, PspA, pneumolysin (PLY), PsrP, PhtD pneumococcal histidine triad protein D, conjugate vaccine, pneumococcal protein vaccine, CbpA

## Abstract

*Streptococcus pneumoniae* is an opportunistic pathogen that causes over 1 million deaths annually despite the availability of several multivalent pneumococcal conjugate vaccines (PCVs). Due to the limitations surrounding PCVs along with an evolutionary rise in antibiotic-resistant and unencapsulated strains, conserved immunogenic proteins as vaccine targets continue to be an important field of study for pneumococcal disease prevention. In this review, we provide an overview of multiple classes of conserved surface proteins that have been studied for their contribution to pneumococcal virulence. Furthermore, we discuss the immune responses observed in response to these proteins and their promise as vaccine targets.

## Introduction

*Streptococcus pneumoniae* is a Gram-positive bacterial pathogen that lives as a commensal in the host upper respiratory tract but becomes invasive in the lung, ear, or blood (Bogaert et al., 2004; Weiser, 2010). Due to its high infectivity, *S. pneumoniae (Spn)* can commonly cause invasive pneumococcal diseases (IPDs) such as community-acquired pneumonia (CAP), and sepsis (Kumar et al., 2008; Weiser et al., 2018). Pneumococcal pneumonia is responsible for approximately 14% of all deaths of children under 5 years old worldwide, with the latest World Health Organization metrics reporting 740,180 deaths in 2019 (WHO, 2019; Cilloniz et al., 2016). Acute otitis media (AOM) remains the most common bacterial infection with high incidence rates and the possibility of re-infection for children <5 (Damoiseaux et al., 2006; Liese et al., 2014).

A major virulence factor of *Spn* is the capsular polysaccharide (CPS), which primarily acts as a shield for the bacterium against the host immune system (Geno et al., 2015). The structural identity of repeat units found within the capsular polysaccharide also distinguishes *Spn* into different serotypes, of which there are now over 100 identified (Ganaie et al., 2020; Ganaie et al., 2021). The pneumococcal conjugate vaccines that exist today have been generated utilizing the CPSs, as the immunogens, of the most prevalent serotypes globally associated with IPD and have been very successful at reducing the rates of IPDs for those included serotypes (Wantuch and Avci, 2018; Wantuch and Avci, 2019). However, factors such as geographic distribution of serotypes, serotype replacement or switching, and an increase in antibiotic-resistant strains all contribute to the persistence of IPD as one of the world’s deadliest diseases (Wantuch and Avci, 2018). A first-of-its-kind global assessment of bacterial antimicrobial resistance associated *Spn* with ~600,000 deaths (Murray et al., 2022). Antibiotic resistance is particularly augmented among serotypes included in the vaccines (Gladstone et al., 2019). Another factor to consider is that PCVs cannot protect against the emergence of non-encapsulated *S. pneumoniae* (NE*Spn*) strains found among non-typeable clinical isolates (Park et al., 2012; Park et al., 2014; Mohale et al., 2016). While NE*Spn*’s are yet to be significantly correlated with IPD, they can still result in infections through AOM (Hotomi et al., 2016) or conjunctivitis (Valentino et al., 2014).

In recent years, studies on molecular mechanisms of glycoconjugate vaccine-induced immune responses have shed light on the knowledge-based vaccine design (Avci et al., 2011; Avci et al., 2012; Middleton et al., 2017; Sun et al., 2019). Researchers have found new therapeutic approaches such as capsule-degrading enzymes (Middleton et al., 2018a; Middleton et al., 2018b; Paschall et al., 2020; Wantuch et al., 2021), capsule-specific protective antibodies (Babb et al., 2021; Ozdilek et al., 2021; Huang et al., 2021), or improved conjugation strategies (Duke et al., 2021). However, identifying and testing vaccine candidates employing conserved *Spn* surface protein immunogens remain an important area of research with the goal of serotype-independent coverage. Recognition of a conserved immunogen across pneumococcal isolates, commonly referred to as a serotype-independent vaccine, would allow for a wider breadth of coverage against pneumococcal pneumonia but would also provide better options for protection against AOM or meningitis. Herein, we describe an up-to-date view on four different classes of the well-studied pneumococcal surface proteins by their function and virulence mechanisms, procured immune response in the host as an immunogen, and potential as vaccine targets (**Table 1**).

**Table 1:** Major *Spn* surface protein immunogens.

Protein	Class	Major Virulence Mechanisms	Representative references
PLY	CDC	Cell Death, MRC-1 binding	(Gonzalez-Juarbe et al., 2015; Riegler et al., 2019; Subramanian et al., 2019; Nishimoto et al., 2020)
PspA	CBP	Inhibits complement, binds to lactoferrin, can bind lactate dehydrogenase	(Tu et al., 1999; Hammerschmidt et al., 1999; Hakansson et al., 2001; Senkovich et al., 2007; Mukerji et al., 2012; Park et al., 2021a)
CbpA	CBP	Inhibits complement, binds to secreted IgA, can bind lactate dehydrogenase	(Hammerschmidt et al., 1997; Haleem et al., 2019; Park et al., 2021a)
PcpA	CBP	Pneumococcal adhesion, biofilm formation	(Sanchez-Beato et al., 1998; Glover et al., 2008)
LytA	CBP	Facilitates release of PLY, inhibits complement	(Canvin et al., 1995; Ramos-Sevillano et al., 2015)
LytB	CBP	Biofilm formation, adherence	(Ramos-Sevillano et al., 2011; Bai et al., 2014)
LytC	CBP	Biofilm formation, adherence	(Ramos-Sevillano et al., 2011)
CbpD	CBP	Assists LytA in cell wall degradation	(Kausmally et al., 2005)
CbpE	CBP	Binds human plasminogen	(Attali et al., 2008a)
CbpF	CBP	Unclear, associated with cell wall hydrolases	(Molina et al., 2009)
CbpG	CBP	Pneumococcal adhesion, secreted form can cleave host extracellular matrix	(Mann et al., 2006)
CbpM	CBP	Binds fibronectin	(Afshar et al., 2016)
PhtA	PHT	Inhibits complement	(Ogunniyi et al., 2009)
PhtD	PHT	Inhibits complement	(Ogunniyi et al., 2009)
PhtE	PHT	Inhibits complement	(Ogunniyi et al., 2009)
PsrP	SRRP	Biofilm formation, lung cell adherence	(Rose et al., 2008; Sanchez et al., 2010)

## Cholesterol-Dependent Cytolysin (CDC)

The CDCs are a family of pore-forming toxins expressed by a multitude of bacterial species and have been shown to have additional and important effects besides their primary activity as beta-hemolytic toxins (Tweten, 2005). The only CDC expressed by *Spn* is pneumolysin (PLY).

### PLY

Like other CDCs, PLY contains the threonine-leucine amino acid motif that mediates cholesterol recognition and membrane binding (Farrand et al., 2010). Multiple PLY monomers congregate at cholesterol lipid rafts of a cell to form the pre-pore complex before inserting themselves into the lipid bilayer (Nishimoto et al., 2020). PLY is unique among the CDCs because it has lost its signal peptide for the type II secretion pathway and it incorporates a domain that can activate the classical complement pathway and promote inflammation (Paton et al., 1984). PLY induces necroptosis (Gonzalez-Juarbe et al., 2015; Riegler et al., 2019), apoptosis, and direct cell toxicity (Nishimoto et al., 2020) acting on a plethora of cell types (Beurg et al., 2005; Braun et al., 2007). Another function of PLY is binding to the host receptor, mannose receptor C type-1 (MRC-1), to downregulate inflammation and enhance bacterial survival in the airways (Subramanian et al., 2019). A detailed discussion of PLY pathogenicity can be found here (Nishimoto et al., 2020).

PLY has been studied as a potential vaccine candidate going back decades (Paton et al., 1983; Anderson and Feldman, 2017), as it is present in practically all infectious strains (Kanclerski and Mollby, 1987). In mice, monoclonal antibodies against PLY decreased bacterial burden in the lung and protected against invasive disease (Garcia-Suarez Mdel et al., 2004). There is evidence for anti-PLY antibodies helping delay pneumococcal carriage in high-risk infants (Francis et al., 2009) and protecting healthy individuals against pneumococcal infections (Huo et al., 2004). In mouse immunization experiments, PLY first showed promise when it protected against multiple *Spn* serotypes (Alexander et al., 1994). However, PLY still exhibited hemolytic activity in host cells, even when constructed to have a reduced cytotoxicity (Baba et al., 2001). Different derivatives of a detoxified PLY mutant (dPLY), a recombinant pneumolysoid with the intrinsic cytolytic functionality removed, have shown reactivity to IgGs against the crucial PLY epitopes and have been used successfully as an immunogen for specific serotypes in mice and rhesus macaques (Garcia-Suarez Mdel et al., 2004; Kirkham et al., 2006; Denoel et al., 2011; Salha et al., 2012). Further, chemically detoxified PLY is another derivative that induced anti-PLY IgG response with no sign of tissue damage in host histopathology exams and protected against intranasal challenge across three distinct serotypes (Hermand et al., 2017). More recently, multiple studies have successfully used dPLY or immunogenic regions of PLY as a standalone vaccine candidate to confer protection from *Spn* isolates (Thanawastien et al., 2021). dPLY has also been tested in conjunction with other immunogenic proteins as broadly protective vaccines that have made it up to phase II trials (Feldman and Anderson, 2014; Chen et al., 2015)(Clinical Trial Number, NCT01262872) (**Table 2**), or as a complementary immunogenic boost for the PCVs (Rowe et al., 2019).

**Table 2:** Representative candidate protein-based vaccine formulations and trials.

Protein	Phase	Identifier	Status	Representative References
PhtD	Phase I	NCT01767402	Completed	(Leroux-Roels et al., 2015)
dPLY, PhtD-dPLY +- PCV10	Phase I	NCT00707798	Completed	(Leroux-Roels et al., 2014)
PhtD-dPLY	Phase II	NCT00896064	Completed	–
PlyD1	Phase I	NCT01444352	Completed	(Kamtchoua et al., 2013)
PhtD	Phase I	NCT01444001	Completed	(Seiberling et al., 2012)
PcpA, PcpA + PhtD	Phase I	NCT01444339	Completed	(Bologa et al., 2012)
PhtD + PcpA + PlyD1	Phase I	NCT01764126	Completed	(Brooks et al., 2015)
		NCT01446926		
PcsB, StkP, PsaA	Phase I	NCT00873431	Completed	–
dPLY + PsaA + 24 CPS	Phase I/II	NCT03803202	Completed	–
	Phase I	NCT04525599	Active	–
PspA + PlyD	Phase Ia	NCT04087460	Active	–
PHiD + dPLY + PhtD	Phase II	NCT01262872	Completed	(Odutola et al., 2016; Odutola et al., 2017; Odutola et al., 2019)

**Figure 1:**
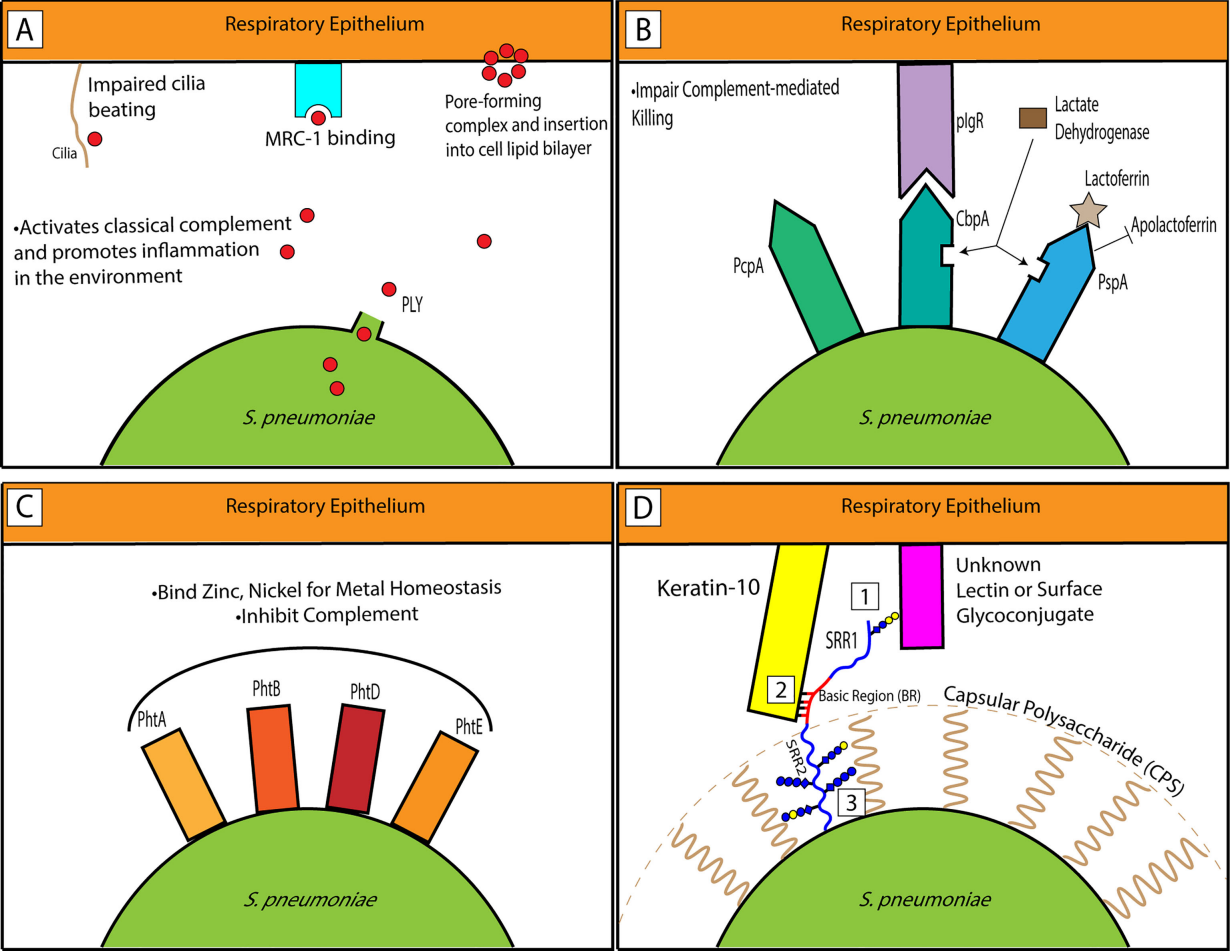
Interactions of pneumococcal factors representative of classes within the host respiratory epithelium. **(A)** PLY is released from *Spn*. It is pro-inflammatory in the environment and PLY monomers aggregate to form ring complexes that can trigger host cell death. It can interact with MRC-1 to promote bacterial survival. **(B)** CBPs impair complement-mediated killing by the host. PcpA influences biofilm formation and pneumococcal adhesion. CbpA binds its main receptor, pIgR, to promote adherence. PspA binds lactoferrin for critical iron uptake for the bacterium from the host and inhibits apolactoferrin. Both CbpA and PspA can bind lactate dehydrogenase to enhance virulence. **(C)** The PHTs are well-conserved surface proteins on the pneumococcus and maintain metal homeostasis through Zn and Ni binding. **(D)** A model showing PsrP’s SRR1-BR region extending past the CPS. While the SRR1 glycans potentially interact with another host ligand (1), the basic region binds Keratin-10 (2). Alternatively, as previously hypothesized, the glycans may be employed as important interactors with the CPS to hoist PsrP up so that it can extend past the capsule (3).

In light of the past and current preclinical research and clinical phase trials, PLY—specifically the recombinant pneumolysoid—has shown significant promise as an important member of the multicomponent protein vaccine approach. PLY’s multitudinous detrimental functions on the host (**Figure 1A**) indicate its potential value as a vaccine target.

## Choline-Binding Proteins (CBPs)

CBPs are a major class of *Spn* surface proteins (Perez-Dorado et al., 2012). Phosphorylcholine residues in repeating units of teichoic and lipoteichoic acids on the cell wall allow CBPs to bind and localize on the surface of *Spn* (Perez-Dorado et al., 2012). CBPs are especially interesting because many orthogonal approaches have unbiasedly reached the same conclusion: most CBPs contribute to bacterial virulence, and are immunogenic targets in human sera exposed to pneumococcal pneumonia (Giefing et al., 2008). Recent advances have been made toward CBPs as candidate antigens in vaccine design as well as expanded knowledge on pathogenicity and virulence mechanisms (Sempere et al., 2021). Here, we review CBPs that have shown contributions to *Spn* pathogenicity and are utilized in preclinical vaccine studies and clinical trial vaccine formulations.

### PspA

Pneumococcal surface protein A, PspA, is one of the most well-studied CBPs. Its vaccine potential was initially identified over three decades ago as a protein expressed in all clinically relevant serotypes (Crain et al., 1990). PspA contains an N-terminal alpha-helical domain (αHD), a proline-rich domain (PRD), and the choline-binding domain at the C-terminus (Hollingshead et al., 2000). PspA is, however, highly variable between serotypes and strains in its αHD, being branched into six different clades based on sequence diversity and organized into three families due to sequence homology between clades (Hollingshead et al., 2000). Although there are two main families (Family 1 and Family 2) that encompass the majority of PspA variability, there is also a Family 3 of more uncommon PspAs as well as clinical isolates that do not express PspA. This has made it a challenge to resolve PspA as a conserved protein. PspA is known to bind to lactoferrin (Hammerschmidt et al., 1999; Hakansson et al., 2001; Senkovich et al., 2007) and has been implicated in both pneumococcal carriage as a colonization factor (McCool et al., 2002) and in invasive disease due to its ability to inhibit complement activation and opsonophagocytosis, the main clearance mechanism by the host immune system (Tu et al., 1999; Ren et al., 2003; Mukerji et al., 2012). However, in a serotype 3 strain and a serotype 19F strain, PspA had the added ability as an adhesin to bind GAPDH present on the surface of dying host cells (Park et al., 2021b). Similarly, PspA has recently demonstrated the ability to bind host lactate dehydrogenase (LDH) to enhance pneumococcal virulence through co-opted metabolic activity (Park et al., 2021a). These findings have culminated in new forms of pathogenicity for PspA and unexplored potential as a vaccine candidate (Lane et al., 2022).

PspA has been investigated for more than three decades as an immunogen and potential vaccine candidate. In a phase I clinical trial, immunizations with a recombinant family 1 PspA produced in *E. coli* and administered to humans showed that PspA was immunogenic, and it passively protected mice against a pneumococcal challenge from serotypes 3, 6A, or 6B (Briles et al., 2000). Later on, a mouse monoclonal antibody specific for PspA induced a robust deposition of complement on the pneumococcal surface indicating a mechanism for PspA specific antibody responses (Ren et al., 2004). Because of its high variability, there was concern that many PspA variants would not be covered by a vaccine; however, recent research has elucidated the proline-rich domain (PRD) of PspA, which is far more conserved, could be used for uncommon strains outside of family 1 or family 2 (Mukerji et al., 2018). Thus, a strong collaboration between a subset of studies has culminated in a trivalent version of PspA that aims to redundantly cover all families and clades of PspA (Nakahashi-Ouchida et al., 2021). Macaques immunized with this trivalent PspA vaccine had high IgG titers towards family 1 or 2 strains and had reduced bacterial load in the lungs compared to control macaques following an intratracheal challenge from a family 1 and a family 2 PspA strain. PspA is in an active clinical trial as a multicomponent vaccine with PlyD (Clinical Trial Number, NCT04087460)(**Table 2**). While the variability in PspA remains a challenge, it continues to be a strong candidate with decades of research behind it.

### CbpA/PspC

Choline-binding protein A, CbpA, was first identified through its homology with PspA (McDaniel et al., 1992), but unconnectedly found as a pneumococcal protein that binds secretory IgA (SpsA) (Hammerschmidt et al., 1997) and as a novel CBP for its role in adherence and immunogenicity (Rosenow et al., 1997). Because of its unique set of roles, it is viewed as the major adhesin in the pneumococcus. It is also highly polymorphic, with 11 different group classifications based on homology and protein sequence among pneumococcal isolates (Brooks-Walter et al., 1999). This antigenic variation in the protein has recently been indicated as an immune evasion mechanism for the bacterium (Georgieva et al., 2018). CbpA has established roles in nasopharynx colonization and pneumococcal pneumonia (Balachandran et al., 2002), but is more notably associated with meningitis (Orihuela et al., 2009). CbpA can bind polymeric immunoglobulin receptor (pIgR), which enables the bacterium to translocate across human nasopharyngeal epithelial cells (**Figure 1B**) (Zhang et al., 2000). The binding of CbpA to host protein factor H inhibits the alternative complement pathway (Weiser et al., 2018).

CbpA, similar to PspA, diminishes the effects of complement-mediated killing (Haleem et al., 2019), however, antibodies against CbpA have been shown to activate complement deposition and increase opsonophagocytosis (Ricci et al., 2011). CpbA and PspA have also recently been shown to have similar potential for binding lactate dehydrogenase released from dying host cells enhancing virulence of the bacterium in damaged tissues (Park et al., 2021a). As a vaccine candidate, CbpA has shown the ability to confer protection as the sole immunogen (Ogunniyi et al., 2007; Cao et al., 2009). Anti-CbpA antibodies are induced through help from CD4+ T lymphocytes following immunization with CbpA (Cao et al., 2009). However, CbpA has also been coupled with many other pneumococcal immunogens and the responses are additive and longer-lasting (Ogunniyi et al., 2007). One of the combinations involves the above-mentioned pneumolysoid and CbpA combined as a fusion protein, which has shown promise as a vaccine with broad protection potential in mouse models of immunization, colonization, and infection (Mann et al., 2014).

### PcpA

Pneumococcal choline-binding protein A, also sometimes referred to as CbpN, is another promising vaccine candidate that has emerged. First elucidated as a pneumococcal adhesion protein due to its leucine repeat regions (LRRs) (Sanchez-Beato et al., 1998), it was a hit in the genome-wide mutagenesis screen for lung virulence factors (Hava and Camilli, 2002) and subsequently shown to provide protection in lung infection and sepsis (Glover et al., 2008). Importantly, PcpA is present in all clinically relevant strains tested so far which encompassed 25 different strains (Glover et al., 2008). It has also been shown to be present in over 90 percent of pneumococcal clinical isolates ranging from non-complicated pneumonia to meningitis (Selva et al., 2012).

The virulence of PcpA is attributed to its pneumococcal adhesion and biofilm formation functionalities (Glover et al., 2008). Interestingly, while PcpA elicits protection in the lower respiratory tract, it does not impede colonization of the upper respiratory tract (Glover et al., 2008). PcpA-specific mAbs promote complement C3 deposition and are macrophage-dependent, as removal of either complement or macrophages compromised protection in mice (Visan et al., 2018). For these reasons, there have been multiple vaccination studies involving PcpA (Verhoeven et al., 2014; Verhoeven et al., 2014; Xu et al., 2015; Brooks et al., 2015; Ochs et al., 2016). In one study, PcpA was a component of a trivalent pneumococcal protein vaccine in a mouse model of immunization. PcpA-specific booster serum responses contributed to the clearance of pneumonia and reduced inflammation and tissue damage in the lungs (Xu et al., 2015). These IgG and IgA boosts from the trivalent protein vaccine, specifically, have also shown a significant reduction in AOM in children (Xu et al., 2017). Due to its demonstrated safety and immunogenic potential against IPD and AOM, PcpA continues to be a mainstay in pneumococcal vaccine formulations in preclinical and clinical studies. PcpA is tested as a vaccine candidate in multiple clinical trials (**Table 2**).

### LytA, LytB, LytC

*N*-acetylmuramoyl-L-alanine amidase (LytA), *N*-acetylglucosaminidase (LytB), and LytC, an autolytic lysozyme involved in polysaccharide cleavage on *N*-acetylmuramoyl-*N*-glucosaminyl residues, comprise a family of cell wall hydrolases (CWH) with established roles in virulence and observed potential for protection.

LytA is the more well-studied of the three enzymes due to its role in lysis being associated with the release of the cytotoxin PLY (Canvin et al., 1995). LytA, independent of its linkage to PLY, has shown an ability to inhibit activation of the alternative and classical pathways of the complement system (Ramos-Sevillano et al., 2015). The same study shows that LytA can cleave the C3b and iC3b components of complement-mediated opsonophagocytosis, indicating its role in evasion of the host immune response (Ramos-Sevillano et al., 2015). Following intranasal immunization with LytA, mice elicited both IgG and IgA antibodies that conferred protection against intranasal and intraperitoneal challenges from strains in serotypes 23F, 19F, 6A, 14, and 6B (Yuan et al., 2011).

LytB has been characterized as a CWH that positions itself at the polar regions on the surface of *Spn* and facilitates localized peptidoglycan hydrolysis and distinct cell separation after multiplying (De Las Rivas et al., 2002). It contributes toward biofilm formation and attachment to host epithelial cells, helps avoid complement-mediated immunity, and is well-conserved (Moscoso et al., 2005; Ramos-Sevillano et al., 2011; Bai et al., 2014). Immunizations with LytB in mice showed a strong IgG response in subclasses IgG1, IgG2b, and IgG3 (Corsini et al., 2016). This study also showed that antibodies against LytB increased recognition towards C1q and C3b to assist classical complement pathway activation and LytB-immunized mice were significantly better protected after challenge with strains in serotypes 23F and 3 (Corsini et al., 2016).

LytC was independently discovered through the same genome wide identification effort of proteins that protected mice from lethal sepsis challenge in which LytB was found (Wizemann et al., 2001). It has been established in similar roles to LytB for nasopharynx colonization and biofilm formation (Gosink et al., 2000; Ramos-Sevillano et al., 2011). However, direct immunization with LytC or antibodies directed against LytC have not as yet been published.

### CbpD, CbpE, CbpF, CbpG, CbpM

CbpD has been previously described as a murein hydrolase that works with LytA to degrade the pneumococcal cell wall of cells during the competence (Kausmally et al., 2005). CbpE has been reported as an important receptor for human plasminogen, the precursor of plasmin, and this interaction is intrinsic for pneumococcal dissemination into host tissues (Attali et al., 2008a; Attali et al., 2008b). CbpF is one of the more abundant proteins of the *Spn* surface, is associated with CWHs like LytA, LytB, and LytC, and has been successfully crystallized (Molina et al., 2007; Molina et al., 2009). These crystal structure studies have resulted in a molecular understanding of esters of bicyclic amines (EBAs) and how they may be used to compete with CBP binding to teichoic acid as a new generation of drugs aimed at limiting pneumococcal growth (Silva-Martin et al., 2014). CbpG was previously identified as a CBP involved in pneumococcal virulence with multifunction as a surface protein for adhesion and as a secreted form that can cleave host extracellular matrix (Gosink et al., 2000; Mann et al., 2006; Attali et al., 2008a). CbpG has been assessed as a protective antigen, where vaccinated mice developed antibodies against CbpG and showed resistance to pneumococcal colonization and statistically significant protection against bacteremia (Mann et al., 2006). CbpM has recently been shown to interact with fibronectin and was recognized in the sera acquired from ten patients with pneumococcal pneumonia (Afshar et al., 2016). CbpG and CbpM also worked well recently as immunogens to protect mice from a serotype 19F challenge (Kazemian et al., 2018).

To sum up, CBPs discussed above have shown vaccine potential in mouse immunization studies and/or human clinical trials. A diverse array of host interactions has been elucidated through pneumococcal adhesion properties associated with different CBPs (**Figure 1B**). PspA virulence has long been linked to its binding to lactoferrin. More recently, it has also been shown to bind host GAPDH and LDH. CbpA binds pIgR and sIgAs. PcpA is an important pneumococcal adhesion protein. Antibodies against any of these targets to neutralize these interactions can serve to weaken the virulence of the bacterium and allow the host immune response to take effect. Difficulties of CBPs as candidate vaccines may come from their sequence variability towards the N-termini, the region that is surface-exposed. While important for pneumococcal virulence, currently none of the proteins discussed in the ‘Other CBPs’ category have been included in any clinical trial (**Table 2**).

## Pneumococcal Histidine Triad Proteins (PHTs)

The pneumococcal histidine triad proteins (PHTs) are a set of surface proteins in the *Streptococcus* genus with a role in metal ion homeostasis and a category-defining HxxHxH protein motif (Plumptre et al., 2012). They were first discovered based on their putative hydrophobic leader sequences, which indicate they were transported across the cytoplasmic membrane and thus were surface-exposed (Adamou et al., 2001). Large-scale mutagenesis screenings for *in vivo* lung infection also showed reduced fitness in bacteria with mutations in *phtA*, *phtB*, or *phtD* (Hava and Camilli, 2002). Including *phtE*, another member of this group, these four gene products redundantly bind and transport zinc (Plumptre et al., 2014). More recently, they have been observed to be highly upregulated in the presence of nickel (Manzoor et al., 2015) and a peptide-based approach revealed a strong binding potential to nickel as well (Miller et al., 2018). All four PHTs are necessary components for pneumococcal survival and proliferation in the nasopharynx (Plumptre et al., 2014). Like PspA above, PHTs inhibit the complement pathway (**Figure 1C**) (Ogunniyi et al., 2009).

### PhtA, PhtB

Pneumococcal histidine triad protein A (PhtA) was the first identified histidine repeat protein in *Spn.* Its sequence was then used to find homologous ORFs, which resulted in the characterization of the PHT family (Adamou et al., 2001). PhtA is highly conserved among the 23 serotypes tested that comprise the PPSV23 multivalent polysaccharide vaccine. Interestingly it was noted that for certain serotypes, PhtA and PhtB have gone through recombination events that have resulted in the resemblance of a fusion protein (Adamou et al., 2001). This has been observed again recently as a PhtA/PhtB fusion protein and a PhtA/PhtD fusion protein (Kawaguchiya et al., 2019). PhtB is also well conserved when it is on its own and not fused with PhtA, sharing 87% sequence homology with PhtD (Adamou et al., 2001). Immunizations with full-length PhtA or full-length PhtB have shown reactive mouse antisera to specific pneumococcal isolates. However, only full-length PhtA bound to all PPSV23 serotype bacterial isolates observed by flow cytometry and conferred protection in mice sepsis challenge with serotype 6A (Adamou et al., 2001). Furthermore, PhtA was present when tested in convalescent sera of 5 infants (Adamou et al., 2001). These findings convey the immunogenicity of this protein as natural to the host response. Immunization in mice with recombinant PhtB conferred protection against serotype 3 intranasal pneumococcal challenge (Zhang et al., 2001). Although they are well conserved and immunogenic, PhtA and PhtB or their fusion proteins have not yet been tested in clinical settings.

### PhtD

Pneumococcal histidine triad protein D (PhtD) is the most studied of the PHTs. In one study, immunizations with PhtD protected mice against a serotype 6A sepsis challenge, as well as against a serotype 4 strain and the highly virulent serotype 3 strain, WU2 (Adamou et al., 2001). Purified human serum IgG antibodies recognizing PhtD have been further described, causing a significant reduction of pneumococcal adherence to human airway epithelial cells (Khan and Pichichero, 2012). Furthermore, intranasal immunization with PhtD generated robust antiserum and protected mice against pneumococcal colonization (Khan and Pichichero, 2013). PhtD has gone through phase I trials as a standalone immunogen or in combination with PcpA, showing efficacy at all dosages with no observed adverse reactions (**Table 2**) (Seiberling et al., 2012; Bologa et al., 2012). Recently, it was shown that antibodies against PhtD facilitated pneumococcal clearance through a complement- and macrophage-dependent opsonophagocytosis (Visan et al., 2018). In another study, immunizations of mice with PhtD and a C-terminal fragment of PhtD induced robust humoral immunity protecting mice against pneumococcal challenge (Andre et al., 2021). In sum, PhtD has emerged in serotype-independent vaccine design due to its robust immune response and feasibility for inclusion in multicomponent vaccine trials.

### PhtE

Pneumococcal histidine triad protein E (PhtE) differs from the other PHTs in the family because it has an extra histidine repeat region and it only shares 32% homology with its other family members (Adamou et al., 2001). Immunoblotting with antibody against recombinant PhtE recognized a full-length version as well as a potential mature, truncated version of PhtE in *Spn* whole cell lysates (Adamou et al., 2001). Further work has observed similar results to PhtD, with antibodies against PhtE reducing pneumococcal adherence and protecting mice against colonization (Khan and Pichichero, 2012; Khan and Pichichero, 2013). PhtE, also known as BVH-3, was used to immunize mice and confer protection against pneumococcal challenge (Hamel et al., 2004). PhtE has not yet been evaluated in clinical phase trials.

Of the PHTs, PhtD has shown high promise as a vaccine candidate with efficacy in preclinical animal studies and early human trials. Although immunizations of mice with PhtA, PhtB and PhtE have conferred variable degrees of protection against *Spn* challenge, PhtD had a significantly stronger and larger serotype coverage for the same protection, and it showed significantly higher reactivity against convalescent human sera collected from infants with culture-proven pneumococcal bacteremia (Adamou et al., 2001). Because the C-terminal region of PhtD is sufficient as a robust immunogen, it adds to its potential for inclusion in multicomponent vaccine design. This has been evident in multiple clinical trials (**Table 2**).

## Serine-Rich Repeat Proteins (SRRPs)

SRRPs are a unique group of very large, glycosylated, surface-exposed proteins in Gram-positive bacteria and are commonly found within the *Streptococcus* genus (Lizcano et al., 2012). They fall in the category of LPXTG proteins, a motif that anchors the protein to the cell wall (Siegel et al., 2017). Because of their large size and abundant glycosylation, they require a dedicated accessory Sec system for transportation and export (Rigel et al., 2009). They are usually categorized as cell adhesins and have been strongly implicated in bacterial virulence from their functional binding (Wu et al., 1998; Siboo et al., 2005; Xiong et al., 2008; Sanchez et al., 2010).

### PsrP

In *S. pneumoniae*, the Pneumococcal Serine-Rich Repeat Protein (PsrP) was first identified through large-scale mutagenesis screenings for lung infection (Hava and Camilli, 2002) and subsequently studied as a pathogenicity island (Obert et al., 2006). The PsrP locus is a 37 kB region that contains the protein itself as well as a multitude of specific glycosyltransferases and the associated Sec transportation machinery (Obert et al., 2006). It has a basic region (BR) between two serine-rich repeat regions (SRR1 and SRR2) that binds to Keratin 10 on lung cells (Shivshankar et al., 2009). *S. pneumoniae* mutants deficient in PsrP are unable to establish lung infection in murine mouse models (Rose et al., 2008). Keratin-10 as a ligand is elevated in cellular senescence of lung cells and these keratin-10/PsrP interactions have been linked to increased susceptibility to pneumococcal pneumonia in the elderly (Shivshankar et al., 2011; Lizcano et al., 2012). Importantly, PsrP is also integral for biofilm formation (Sanchez et al., 2010; Schulte et al., 2016), a major factor in the bacterium’s ability to colonize a host and aggregate (Sanchez et al., 2010; Blanchette-Cain et al., 2013).

The mature glycan of an SRRP has been discovered in a different *Streptococcus* species but has yet to be completely and directly elucidated for PsrP (Zhu et al., 2016). Like other SRRPs, PsrP is predicted to be heavily O-glycosylated due to the plethora of serine and threonine residues in the SRR1 and SRR2 regions. GtfA, the glycosyltransferase responsible for adding the first GlcNAc moiety in many SRRPs, was documented to function similarly for PsrP (Shi et al., 2014). GtfA deficient mutants also demonstrated an attenuation of biofilm formation, lung adhesion, and virulence in mice (Lizcano et al., 2017). The second, third, and fourth steps of the PsrP glycosylation pathway have also been elucidated and a proposed model of the complete glycosylation pathway was established (Zhou et al., 2010; Jiang et al., 2017). Recently, however, multiple glycosyltransferases (GTs), including some as-of-yet uncharacterized, were shown to significantly impact PsrP’s biofilm formation and adhesion properties as well as lung infection in mice by intratracheal challenge (Middleton et al., 2021).

PsrP is predicted to be present in approximately 50% of all clinical isolates of *Spn* (Munoz-Almagro et al., 2010; Selva et al., 2012). Antibodies against a recombinant portion of PsrP containing the SRR1 and BR regions (rPsrP) were able to neutralize bacterial adhesion to lung cells *in vitro* and passive immunization with rPsrP rabbit antiserum provided protection *in vivo* to mice against challenge (Rose et al., 2008). Although antibodies and passive immunization have shown protection against bacterial lung infection, there isn’t established work on PsrP as a vaccine for direct immunizations. It is possible that the glycosylation of PsrP in its native state is an important component of its antigenicity to be understood and harnessed (**Figure 1D**).

## Discussion

When discussing pneumococcal protein vaccine candidates, it is important to emphasize the success of the PCVs in drastically reducing mortality from pneumococcal disease. For a large amount of IPD associated with the serotypes included, the PCVs are a major success story (Avci et al., 2019). However, it is also important to recognize the challenges and complexity of continuing this empirical, serotype-specific approach for emerging serotypes and unencapsulated isolates (Duke and Avci, 2018; Avci et al., 2019). As the diversity of *Spn* serotypes in clinical isolates continues to increase, higher valency of PCVs will be required. In addition, the possibility of carrier-induced epitope-specific suppression and the known variable humoral response elicited among serotypes included in multivalent preparations represent some of the challenges in the development of conjugate vaccine preparation (Di John et al., 1989; Avci et al., 2019). Thus, current research is exploring many forms of protein vaccine candidates as individual elements, together as multi-component protein vaccines, and a hand-in-hand complementary approach with the PCVs. A conserved pneumococcal vaccine or (to put it ambitiously) a universal pneumococcal vaccine goal has fueled protein-based vaccine research for many decades.

While this review focuses on surface protein candidates isolated from pneumococcus, there are two alternative approaches that have made considerable progress. The first is an inactivated whole-cell vaccine of a non-encapsulated pneumococcal strain that has progressed through pre-clinical trials, showing protection against nasopharyngeal carriage as well as IPD (Keech et al., 2020). Currently, forty-two human patients had safe responses and demonstrated antigen-specific antibodies and T-cell cytokine responses (Keech et al., 2020). The second approach uses a modified, live-attenuated *S. typhimurium* strain as a carrier to deliver the pneumococcal antigen PspA (Zhang et al., 2011). This vector system has shown significant protection in mice with high levels of sIgAs in the nasal mucosa, high IgG levels in serum, and is moving into phase I trials (Zhang et al., 2011; Liu et al., 2020).

Spn surface protein immunogens reviewed have shown variable degrees of protection capacity in preclinical and clinical studies making them candidates for next-generation pneumococcal vaccine formulations. Interestingly, the problems with serotype distribution based on geographical location can impact a protein-based vaccine too. A study using PCR detection of many of the discussed pneumococcal surface proteins observed non-uniform presence of multiple proteins originally expected to be conserved (Blumental et al., 2015), while another study showed downregulation of PcpA and PhtE and no changes in PLY, PspA, or PhtD when comparing serotypes 15A and 35B, two serotypes with increasing incidence rates and outside of PCV coverage (Fuji et al., 2021). Partly due to these reasons, a growing effort has developed for a multi-component approach as opposed to individual proteins in vaccine design (**Table 2**). A multi-component vaccine target comprised of PLY and PhtD together with 10 serotype-specific polysaccharide conjugates as an exciting prospect, is evaluated in phase II trials (Odutola et al., 2017). A trivalent version of three PspA variants has been generated to provide coverage across all families and clades (Nakahashi-Ouchida et al., 2021), while PspA has also been coupled with CbpA and PLY (Chen et al., 2015). Completed and ongoing trials of protein-based vaccines are listed in **Table 2**. Of note, most recently new formulations have shown success in mice, and/or macaques, indicating potential future clinical trials to be initiated. A recent study analyzed 100 pneumococcal proteins for their predicted CD4^+^ T cell immunogenicity, and the prediction results were validated by stimulating human PBMCs (van de Garde et al., 2019). Based on the results of this study, PspA, PLY, and PhtD were indicated as vaccine candidates of interest. The study also noted pneumococcal surface adhesin A (PsaA) and PcsB, a peptidoglycan hydrolase, as potentially important immunogens (van de Garde et al., 2019). While these proteins do not fall into the classes discussed in this review, they are notably present in a unique clinical trial (Clinical Trial Number, NCT00873431). These constructs have all been shown to protect against many clinical isolates, and ongoing preclinical studies and clinical trials will shed light on whether they will reach a similar threshold of efficacy and coverage to PCV13 or the recently launched PCV15 and PCV20.

Considering the virulence mechanisms and current representation in clinical trial vaccine formulations (**Table 2**), dPLY and PhtD show high promise. Stimulating an antibody response against PLY can protect the host from tissue damage that would result in enhanced virulence and sensitive tissue access for the bacterium, specifically in the lung and heart. It also is present in all pneumococcal strains without variability that some of the other proteins display. Similarly, PhtD has a good prevalence among clinical pneumococcal isolates and has demonstrated strong immunogenic properties in convalescent human sera. Besides these two protein immunogens, PspA has shown to be highly immunogenic and protective albeit with its’ high degree of sequence variability in clinical *Spn* isolates. PspA is moving into clinical trials in combination with PLY (**Table 2**) and has shown potential as a trivalent PspA vaccine in macaques.

PsaA—while not a member of any of the classes reviewed above—is a well-studied, conserved lipoprotein expressed by all Spn clinical isolates (Rajam et al., 2008). PsaA is highly immunogenic and it contributes to the pathogenicity of Spn (Rajam et al., 2008). It is a surface-exposed zinc binding member of an ABC-type transport system (Lawrence et al., 1998). It also exhibits adhesin properties and binds to human Annexin A2 on airway epithelial cells (Hu et al., 2021). As an immunogen, PsaA has shown vaccine potential, inducing robust immune responses (Johnson et al., 2002; Oliveira et al., 2006; Whaley et al., 2010). In a murine model of colonization with an *Spn* serotype 19A, concomitant administration of recombinant PsaA and PCV7 reduced colonization (Whaley et al., 2010). In a separate study, nasal inoculation of mice with recombinant lactic acid bacteria expressing PsaA induced a systemic and mucosal immune response and decrease in Spn colonization (Oliveira et al., 2006). Antibodies against PsaA showed a significant reduction in nasopharyngeal colonization in a mouse nasal colonization model (Johnson et al., 2002). PsaA was included in a promising clinical trial vaccine formulations with two other proteins, StkP and PcsB, that were prevalent in human sera of patients recovered from pneumococcal pneumonia (Giefing et al., 2008) (Clinical Trial Number, NCT00873431) (**Table 2**). PsaA is also a vaccine component in two other clinical trials (Clinical Trial Numbers, NCT03803202 and NCT04525599) (**Table 2**).

Sitting mostly on the outside of the pneumococcal protein vaccine conversation has been PsrP. While lung adhesion and biofilm formation are highly important functions for pneumococcal infection and PsrP has a proven link to these functions, vaccination efforts have not been reported. This may be due to the lower overall prevalence of PsrP in clinical isolates compared to PLY and PspA or the molecular difficulties associated with the size and glycosylation state of the protein. The PCVs have brought significant attention to the possibilities involving glycosylation in vaccine design, but PsrP as a glycoprotein has yet to be considered. The potential role of PsrP glycans as part of B and T cell epitopes (Avci et al., 2011; Sun et al., 2016) may indicate PsrP as a protective vaccine target.

In conclusion, there are many attempts ongoing to achieve a serotype-independent, conserved pneumococcal protein vaccine while the serotype-dependent conjugate vaccines keep IPDs under control on a global scale. A serotype-independent protein vaccine has many potential advantages: a potentially lower-cost alternative for children in developing countries, higher coverage of geographical serotypes that can lead to IPD, or a new/alternative line of defense against AOM infection/recurrent infections. Cutting-edge approaches and innovative knowledge-based vaccine designs are making their way through preclinical research and clinical trials. These stepwise advancements from mice to macaques to humans highlight the exciting potential for these different surface immunogens in becoming next-generation vaccines.

## Author Contributions

JA and FA wrote the manuscript. Both authors participated in the editorial process of the manuscript. Both authors have approved the manuscript.

## Funding

This work was supported by National Institutes of Health grants R01AI123383, R01AI152766, and R41AI157287 (FA).

## Conflict of Interest

The authors declare that the research was conducted in the absence of any commercial or financial relationships that could be construed as a potential conflict of interest.

## Publisher’s Note

All claims expressed in this article are solely those of the authors and do not necessarily represent those of their affiliated organizations, or those of the publisher, the editors and the reviewers. Any product that may be evaluated in this article, or claim that may be made by its manufacturer, is not guaranteed or endorsed by the publisher.
